# Editorial: Behavior support for people with dementia

**DOI:** 10.3389/fpsyt.2024.1389668

**Published:** 2024-03-27

**Authors:** Stephen Macfarlane, Susan Kurrle, Sally Grosvenor, Colm Cunningham

**Affiliations:** ^1^ The Dementia Centre, HammondCare, St Leonards, NSW, Australia; ^2^ Faculty of Medicine, Nursing and Health Sciences, Monash University, Clayton, VIC, Australia; ^3^ Faculty of Medicine and Health, The University of Sydney, Darlington, NSW, Australia

**Keywords:** BPSD (behavioral and psychological symptoms in dementia), dementia, interventions, non-pharmacological, person-centered

Acknowledging the complexities of behaviours and psychological symptoms of dementia (BPSD), best practice guidelines and policies advocate for non-pharmacological therapies as the first-line treatment (1).

The Australian government-funded Dementia Support Australia is actively contributing to the understanding and implementation of non-pharmacological interventions. By providing valuable data, this dementia support service plays a pivotal role in assessing the impact of such interventions, aiding in the development of effective strategies to support people with dementia experiencing BPSD (2).

However, significant challenges persist. Failure to recognise unmet needs of the person with dementia, such as pain or adverse environmental stimuli, impacts care quality and individual well-being (3). People living with dementia also face disparities in accessing palliative care services, an inequity particularly striking when compared to those with other life-limiting illnesses such as cancer (4). Moreover, care workers and family members may not recognise the signs that indicate a person with dementia is nearing the end of life, leading to potential lapses in addressing their unique needs during this period.

This Research Topic delves into various dimensions of non-pharmacological interventions, palliative care, and engagement therapy considerations for people living with dementia experiencing BPSD. The articles submitted include study protocols, systematic reviews and meta-analyses, case studies and new research. Palliative care and end-of-life considerations.

In the study titled “*Dying with behavioral and psychological symptoms of dementia in Australian nursing homes: a retrospective case-control study*,” Roach et al. identified factors signalling the risk of death in people experiencing BPSD, and the need for palliative care interventions. This case-control study identified key differences in those who died, including the presence of delirium, drowsiness, reduced oral intake, benzodiazepine use, and nursing concern.

The qualitative study by Juhrmann et al., “*Staff perspectives on end-of-life care for people living with dementia in residential aged care homes*,” examined staff views on providing high quality care for people with dementia living in aged care homes nearing end of life. The findings underscore the need for a person-centred approach to care, enhanced support and education for staff and a conducive work environment. Advance care planning, collaborative multidisciplinary teamwork, and family engagement were also identified as essential components of high-quality palliative care for a person with dementia living in care homes.

## Engagement therapies

Therapeutic benefits of garden use for people living with dementia, including those with BPSD, are well known, revealing improvements in cognitive function, reduced agitation, and enhanced overall well-being through engagement with nature-based environments. The study titled “*The effect of garden use on quality of life and behavioral and psychological symptoms of dementia in people living with dementia in nursing homes: a systematic review*” included 19 publications of quantitative, qualitative and mixed methods, randomized clustered controlled trials, pilot and feasibility studies (Velde-van Buuringen et al.). The findings indicate positive effects of garden use on Quality of Life (QoL) and BPSD in people living in care homes. The review emphasises the importance of addressing physical, social, and organisational aspects within dementia care settings with respect to integration of garden use.

In “*Network meta-analysis of comparative efficacy of animal-assisted therapy vs pet-robot therapy in the management of dementia*,” Du et al included 19 randomised controlled trials (RCTs). In this analysis, agitation was the primary measure and cognitive function and depression were secondary. Pet-robotic therapy emerged as a promising intervention, effectively alleviating agitation in dementia patients compared to controls, while neither animal-assisted therapy or pet-robot therapy resulted in significant improvement in cognitive function or reduction in depression.

The feasibility trial titled “*Can a personalised music listening intervention decrease agitation in hospitalised patients with dementia? A feasibility trial*,” by Lee et al investigates the impact of personalised music on agitation in hospital setting. In this two-way randomised control feasibility study, twenty-one patients received one hour of personalised music daily over a five-day hospital stay. While the impact on decreasing agitation was inconclusive, staff interviews found assisted engagement with patients did not impact clinical care, improved patient mood, and challenged staff mindset around using psychotropic medication to address agitation.

The forthcoming study by Li et al., “*Non-pharmacological interventions for behavioral and psychological symptoms of dementia: A systematic review and network meta-analysis protocol study*,” aims to assess the effectiveness of various interventions, including aerobic exercise, acupuncture, massage therapy, cognitive training, and music therapy. With the goal of identifying optimal therapeutic strategies for BPSD, this study holds the potential to guide future research and clinical practices.

## Multifaceted interventions

Baird et al.‘s paper, “*Clinical impact of a multifaceted intervention aimed at decreasing distress in people living with dementia: evaluating the Reconnect program*,” evaluates the impact of the Reconnect program on engagement, pain, and constipation in a single aged care site. The findings underscored a reduction in distress and psychotropic medication use through staff training, increased staff to patient ratios, supportive environments, and effective management of pain and constipation.

## Complexities within mental health systems

The study by Wolverson et al., “*Family experiences of inpatient mental health care for people with dementia*,” explores the complexities of providing quality care for a person experiencing BPSD in the acute care mental health ward setting. Seven family carers participated in the study, highlighting largely negative experiences during admission. The imperative for enhanced communication, recognition of caregiver expertise, and ongoing research to deepen comprehension of dementia-related mental health admissions becomes evident.

By exploring various approaches, this Research Topic aims to contribute to the evolving landscape of dementia care, fostering a person-centred, non-pharmacological, evidence-based approach for individuals experiencing BPSD. We would like to thank all the authors who contributed to this Research Topic.

## Author contributions

CC: Writing – review & editing. SG: Writing – original draft. SK: Writing – review & editing. SM: Writing – review & editing.
